# A review of autoimmunity and immune profiles in patients with primary ovarian insufficiency

**DOI:** 10.1097/MD.0000000000032500

**Published:** 2022-12-23

**Authors:** Junyu Chen, Shan Wu, Mengqi Wang, Haoxian Zhang, Manhua Cui

**Affiliations:** a Departments of Gynecology and Obstetrics, The Second Hospital of Jilin University, Changchun, China; b Department of Reproductive Endocrinology, Women’s Hospital, Zhejiang University, School of Medicine, Hangzhou, China; c Department of Pharmacy, Xuchang Central Hospital, Xuchang, China.

**Keywords:** autoimmunity, cytokines, oophoritis, ovarian reserve, primary ovarian insufficiency

## Abstract

Primary ovarian insufficiency (POI) is a complicated clinical syndrome characterized by progressive deterioration of ovarian function. Autoimmunity is one of the main pathogenic factors affecting approximately 10% to 55% of POI cases. This review mainly focuses on the role of autoimmunity in the pathophysiology of POI and the potential therapies for autoimmunity-related POI. This review concluded that various markers of ovarian reserve, principally anti-Müllerian hormone, could be negatively affected by autoimmune diseases. The presence of lymphocytic oophoritis, anti-ovarian autoantibodies, and concurrent autoimmune diseases, are the main characteristics of autoimmune POI. T lymphocytes play the most important role in the immune pathogenesis of POI, followed by disorders of other immune cells and the imbalance between pro-inflammatory and anti-inflammatory cytokines. A comprehensive understanding of immune characteristics of patients with autoimmune POI and the underlying mechanisms is essential for novel approaches of treatment and intervention for autoimmune POI.

## 1. Introduction

Primary ovarian insufficiency (POI), previously known as premature ovarian failure, is a clinical syndrome defined as amenorrhea prior to the age of 40 years, accompanied by elevated follicle-stimulating hormone (FSH) (>40 IU/L on two occasions > 4 weeks apart) and decreased estrogen levels.^[1]^ POI is a heterogeneous and multifactorial disorder with a wide spectrum of causes, including autoimmune disorders, genetic alterations, environmental factors and iatrogenic interventions, such as radiotherapy or chemotherapy.^[1]^ Due to ovarian dysfunction, patients often experience menopausal symptoms, including hot flashes, sleep disturbance, loss of libido, urogenital atrophy, dyspareunia, and emotional instability. In addition, patients may also endure various long-term complications related to estrogen deficiency, such as osteoporosis, type 2 diabetes, and cognitive decline.^[2]^

POI affects 1% of women before the age of 40 years and 0.1% of women before 30.^[3]^ Although spontaneous ovulation occurs occasionally, the chance of spontaneous pregnancy in POI patients is merely 5%, and permanent loss of fertility can be observed in most cases of POI.^[4]^ Considering the adverse effects of POI and the urgent desire of POI patients to conceive, POI has been paid global attention especially among young women.

In recent years, emerging studies have shown strong association between POI and autoimmune disorders because of the presence of lymphocytic oophoritis, autoantibodies to ovarian antigens, and concurrent autoimmune diseases. Nevertheless, the exact mechanism of autoimmunity in the etiology of this disorder remains obscure. The goal of this review is to summarize the latest studies regarding the immunological aspects of POI in humans.

## 2. Methods

A literature review was performed using PubMed and Google Scholar up to October 2022, and the keywords including: primary ovarian insufficiency, oophoritis, autoimmunity, cytokines and ovarian reserve. All identified publications were restricted to English only.

## 3. Autoimmunity and ovarian reserve

Human ovaries are susceptible to autoimmune attack, resulting in decreased ovarian reserve and even POI. Researchers recommended that assessment of ovarian reserve should be performed for women with autoimmune disorders, such as Hashimoto thyroiditis, systemic lupus erythematosus (SLE), and type 1 diabetes mellitus (T1DM).^[5]^ A number of predictors for ovarian reserve have been described, including concentration of baseline FSH (bFSH), estradiol, inhibin B, anti-Müllerian hormone (AMH), ovarian volume, and the antral follicle count (AFC).^[6]^ Among these predictors, AFC, AMH and bFSH are typically used to evaluate ovarian reserve, and hence were principally discussed in this section.

### 3.1. Autoimmunity and AMH

Circulating AMH is mainly produced by preantral and small antral follicles in the ovaries.^[7]^ AMH could be served as an excellent predictor for the menopausal transition than other ovarian reserve markers, and the reduction of AMH is the earliest event identified in women who later developed ovarian insufficiency.^[8]^ Therefore, AMH shows a good predictive value for women at risk of POI.

Studies have shown that several autoimmune disorders could impair ovarian reserve, and were related to lower AMH levels (Table 1). For instance, serum AMH levels were lower in women with autoimmune thyroid disease (AITD),^[10]^ which may be due to the presence of thyroid peroxidase antibodies,^[31]^ indicating the association between autoimmune thyroiditis and decreased serum levels of AMH. Interestingly, in adolescents with Hashimoto’s thyroiditis, no impairment of ovarian reserve was found as measured by AMH levels,^[12]^ suggesting that autoimmune disorders may take time to exert detrimental effects on ovarian function.

In addition, impaired ovarian reserve in SLE patients can be observed by detecting AMH levels.^[14,32]^ SLE could cause systemic inflammation including autoimmune oophoritis, and then result in ovarian dysfunction. Moreover, SLE could also lead to disorders of the hypothalamic-pituitary-ovarian (HPO) axis, and subsequent imbalance of hormone ultimately leads to ovarian insufficiency.^[32]^

Given the appearance of autoantibodies prior to symptom onset, T1DM is also known as autoimmune diabetes.^[33]^ It has been found that AMH levels were decreased in mid-aged T1DM women.^[16]^ Interestingly, prepubertal girls with T1DM have higher levels of AMH, suggesting a greater number of follicles in the ovaries during childhood. However, during puberty, the AMH levels of T1DM girls were significantly decreased, indicating the deleterious influence of T1DM on ovarian folliculogenesis is secondary to the increase of gonadotrophin levels in puberty.^[34]^

Rheumatoid arthritis (RA) is another chronic inflammatory autoimmune disease that often occurs in women of childbearing age.^[35]^ Although a few studies failed to find impairment in AMH levels in RA women,^[18,36]^ in other studies, AMH levels were reduced^[19,20]^ and showed a more pronounced decline over time in RA women,^[35]^ suggesting that RA could cause impaired ovarian function.

Furthermore, several other autoimmune diseases, such as psoriasis,^[21]^ dermatomyositis,^[22]^ ankylosing spondylitis,^[23]^ Sjögren’s syndrome,^[24]^ Behçet’s disease, spondyloarthritis^[19]^ and relapsing-remitting multiple sclerosis,^[25]^ have been proven to exert an adverse effect on serum AMH levels. Besides, nonspecific autoimmunity is also negatively associated with AMH levels due to the presence of antiphospholipid antibodies.^[37]^

### 3.2. Autoimmunity and AFC

In in vitro fertilization (IVF), AFC has good predictive value for the number of retrieved oocytes and ovarian response to gonadotrophins. The determination of AFC could be helpful in identifying an appropriate stimulation protocol. Similar to AMH, there has been a lot of evidence for the correlation between autoimmune diseases and AFC declines (Table 1).

**Table 1:** Changes of common ovarian reserve markers in POI compared with controls.

Marker	Article	Disease	Sample size	Patients	Control
AMH (ng/mL)	Saglam et al (2015)^[9]^	AITD (mean ± SD)	167	1.16 ± 0.17*	1.28 ± 0.25
	Magri et al (2015)^[10]^	AITD (median (range))	288	1.40 (1.0–1.7)*	2.27 (1.7–2.3)
	Özalp Akin et al (2018)^[11]^	Hashimoto’s thyroiditis (adolescents) (median (range))	60	1.7 (0.5–5.1)	1.8 (0.29–5.5)
	Pirgon et al (2016)^[12]^	Hashimoto’s thyroiditis (adolescents) (mean ± SD)	60	10.6 ± 10.4*	7.5 ± 7.3
	Tuten et al (2014)^[13]^	Hashimoto’s thyroiditis (median (range))	81	4.82 (1.47–7.38)*	2.44 (1.63–3.29)
	Ma et al (2013)^[14]^	SLE (mean ± SD)	44	1.71 ± 1.29*	3.33 ± 1.76
	Malheiro et al (2014)^[15]^	SLE (median (range))	54	1.23 (0.24–4.63)	1.52 (1.33–1.88)
	Soto (2009) et al^[16]^	T1DM (mean ± SD)	124	12.1 ± 9.2 (pmol/L)	14.2 ± 10.8 (pmol/L)
	Kadiroğullari et al (2020)^[17]^	T1DM (mean ± SD)	107	2.10 ± 1.23 (pmol/L)*	3.05 ± 1.89 (pmol/L)
	Lopez-Corbeto et al (2020)^[18]^	RA (median (range))	119	1.27 (0.42–2.24)	1.31 (0.46–3.09)
	Henes et al (2015)^[19]^	RA (median(range))	Unknown	1.8 (0.0–11.3)*	2.4 (0.3–13.2)
	Brouwer et al (2019)^[20]^	RA (median (range))	Unknown	2.5 (1.54.6)	Unclear
	Aydogan Mathyk et al (2019)^[21]^	Psoriasis (mean ± SD)	72	1.85 ± 1.13*	2.46 ± 1.21
	de Souza et al (2015)^[22]^	Dermatomyositis (n (%))	39	≤1: 8 (50%)*	≤1: 3 (13%)
	Yalçin Bahat et al (2021)^[23]^	Ankylosing spondylitis (mean ± SD)	104	1.188 ± 0.891*	2.203 ± 1.110
	Karakus et al (2017)^[24]^	Sjögren’s syndrome	49	Unclear	Unclear
	Henes et al (2015)^[19]^	Behçet’s disease (median (range))	Unknown	1.1 (0–6.1)*	1.9 (0.1–13.2)
	Henes et al (2015)^[19]^	Spondyloarthritis (median (range))	Unknown	1.5 (0.1–7.4)*	2.3 (0.05–13.2)
	Thöne et al (2016)^[25]^	Relapsing-remitting multiple sclerosis (mean ± SD)	134	2.47 ± 0.26*	3.34 ± 0.34
AFC (n)	Ma et al (2013)^[14]^	SLE (mean ± SD)	44	10.90 ± 3.00*	14.61 ± 4.93
	Ulug et al (2014)^[26]^	SLE (mean ± SD)	40	9.0 ± 1.12*	11.4 ± 1.89
	Malheiro et al (2014)^[15]^	SLE (median (range))	54	6.44 (4.19–7.69)	7.5 (6.03–8.09)
	Kadiroğullari et al (2020)^[17]^	T1DM	107	Unclear	Unclear
	Yamakami et al (2014)^[27]^	primary antiphospholipid syndrome (%)	≤5: 42	≤5: 37	9
	de Souza et al (2015)^[22]^	Dermatomyositis (mean ± SD)	39	10.5 ± 5.6	17.3 ± 10.7
	Yalçin Bahat et al (2021)^[23]^	ankylosing spondylitis (mean ± SD)	104	9.54 ± 2.50*	10.67 ± 1.81
	Oner et al (2013)^[28]^	FMF (median (range))	60	12.0 (11–14)*	14.5 (2–18)
	Karakus et al (2017)^[24]^	Sjögren’s syndrome	49	Unclear	Unclear
	Pirgon et al (2016)^[12]^	Hshimoto thyroiditis (mean ± SD)	60	11 ± 7.2	13.4 ± 5.8
	Tuten et al (2014)^[13]^	Hshimoto thyroiditis (mean ± SD)	81	6.84 ± 3.81	6.08 ± 2.72
bFSH (IU/L)	Ma et al (2013)^[14]^	SLE (mean ± SD)	44	5.18 ± 1.52	4.85 ± 1.00
	Malheiro et al (2014)^[15]^	SLE (median (range))	54	6.44 (4.19–7.69)	6.44 (4.19–7.69)
	Ulug et al (2014)^[26]^	SLE (mean ± SD)	40	8.7 ± 2.60*	6.8 ± 1.50
	Medeiros et al (2009)^[29]^	SLE (median (range))	60	4.6 (0.65–8.17)*	3.4 (0.5–16.7)
	Magri et al (2015)^[10]^	AITD (median (range))	288	9.57 (8.1–11.0)*	7.96 (7.4–8.4)
	Saglam et al (2015)^[9]^	AITD (mean ± SD)	167	7.3 ± 3.6	7.5 ± 3.1
	Pirgon et al (2016)^[12]^	Hashimoto’s thyroiditis (adolescents) (mean ± SD)	60	5.01 ± 2	5.09 ± 1.7
	Tuten et al (2014)^[13]^	Hashimoto’s thyroiditis (mean ± SD)	81	7.16 ± 2.29	6.53 ± 2.55
	Yalçin Bahat et al (2021)^[23]^	ankylosing spondylitis (mean ± SD)	104	6.72 ± 1.14	7.21 ± 1.22
	Karakus et al (2017)^[24]^	Sjögren’s syndrome (mean ± SD)	49	5.5 ± 2.7	4.8 ± 2.1
	Yamakami et al (2014)^[27]^	primary antiphospholipid syndrome (median (range))	42	6.1 (3.8–20.1)	5.8 (2.2–14.4)
	de Souza et al (2015)^[22]^	Dermatomyositis (mean ± SD)	39	6.2 ± 2.0	6.6 ± 3.8
	Aydogan Mathyk et al (2019)^[21]^	Psoriasis (mean ± SD)	72	6.03 ± 2.94	7.10 ± 3.57
	Soto et al (2009)^[16]^	T1DM (mean ± SD)	124	6.2 ± 3.4	7.4 ± 7.2
	Codner et al (2007)^[30]^	T1DM (among PCOS) (mean ± SD)	37	5.1 ± 1.8	5.3 ± 1.7
	Kadiroğullari et al (2020)^[17]^	T1DM (mean ± SD)	107	5.53 ± 1.99	6.00 ± 3.12
	Oner et al (2013)^[28]^	FMF (median (range))	60	10.0 (9-11)*	9.0 (7–9)

AFC = antral follicle count, AITD = autoimmune thyroid disease, AMH = anti-Müllerian hormone, bFSH = baseline FSH, FMF = familial Mediterranean fever, POI = primary ovarian insufficiency, SLE = systemic lupus eryth, T1DM = type 1 diabetes mellitus.

* *P* < .05.

A significant reduction of AFC was confirmed in patients with SLE,^[14,15,26]^ T1DM,^[17]^ primary antiphospholipid syndrome,^[27]^ dermatomyositis,^[22]^ ankylosing spondylitis,^[23]^ familial Mediterranean fever (FMF)^[28]^ and Sjögren’s syndrome.^[24]^ Besides, the duration of psoriasis was negatively associated with AFC, suggesting the contribution of psoriasis to the impairment of ovarian reserve.^[21]^ However, in women diagnosed with Hashimoto’s thyroiditis (including adolescent girls), AFC remained unchanged.^[12,13]^ Moreover, unlike AMH, neither TPO positivity nor thyroid function is associated with AFC.^[38,39]^ These evidences weakened the pathological effects of AITD on ovarian reserve.

### 3.3. Autoimmunity and bFSH

In assisted reproductive techniques (ART), elevated bFSH levels are associated with lower oocyte production in IVF and poor response to ovarian stimulation. However, the relationship between autoimmune diseases and bFSH is weaker than AMH and AFC, and even conflicting. For example, in patients with SLE, bFSH levels increased in others,^[26,29]^ whilst remained stable in some studies.^[14,15]^ Similarly, although it is elevated in sub-fertile AITD women,^[10]^ in most studies, neither thyroid antibody positivity nor thyroid function were related to bFSH concentration.^[38–40]^ Beside AITD,^[9,12,13]^ there was no significant difference between patients with several other autoimmune diseases and those without, including ankylosing spondylitis,^[23]^ Sjögren’s syndrome,^[24]^ primary antiphospholipid syndrome,^[27]^ dermatomyositis,^[22]^ psoriasis^[21]^ and T1DM.^[16,30]^ Only women with FMF had remarkably higher concentrations of bFSH.^[28]^

## 4. Autoimmune disorders in women with POI

As mentioned above, human ovaries are subject to autoimmune attacks, resulting in ovarian dysfunction, including POI. Many studies have elucidated the close relationship between autoimmunity and POI, which is manifested in the presence of lymphocytic oophoritis and ovarian autoantibodies, as well as the concurrence of POI with other autoimmune disorders.^[41–44]^

### 4.1. Autoimmune oophoritis in POI

Autoimmune oophoritis was first described in the cases of Addison’s disease,^[3]^ and approximately 10% women with Addison’s disease have POI.^[45]^ These findings were potent support for the hypothesis of autoimmune etiology of POI. Interestingly, POI frequently precedes Addison’s disease, sometimes even 8 to 14 years earlier,^[3,46]^ which implied that the occurrence of autoimmune oophoritis, POI, and Addison’s disease might be regulated by the same factors. Furthermore, autoimmune polyglandular syndrome (APS) is a series of disorders defined as autoimmunity against endocrine organs, which is closely associated with autoimmune oophoritis.^[44]^ The main features of APS-I (APS type I) included Addison’s disease and ovarian failure.^[47]^ APS-II is related to early gonadal failure in 5-50% of patients, and ranging from 10% to 25% of patients with APS-II will go on to develop POI.^[48]^ Autoimmune oophoritis associated with APS-I or APS-II is responsible for 2% to 10% of POI cases.^[49]^ APS-III is one of the most common co-syndromes of POI, with the prevalence of approximately 33% in POI patients.^[3]^ These results above indicated that POI was strongly correlated with Addision’s disease and APS, and could also be considered as an autoimmune disease.

Autoimmune diseases are featured by the autoreactive T cells and the presence of organ and non-organ-specific autoantibodies.^[50]^ By ovary biopsy, autoimmune oophoritis could be diagnosed with histological inflammatory features, including infiltration of mononuclear inflammatory cells (such as natural killer [NK] cells, macrophages, plasma cells and lymphocytes) in the theca interna of growing follicles.^[51–55]^ However, the absence of inflammatory infiltrates could not completely exclude the possibility of autoimmune mechanisms. Follicular depletion may represent the final stage of autoimmune diseases. At this stage, follicular supply has been exhausted, and presumably target antigens in ovaries have also been eliminated.^[43]^ Therefore, the negative infiltrates may be the consequence of autoimmune etiology, but this conclusion needs more systematic studies, which is challenging due to the fact that the procedure of ovary biopsy is invasive.

The existence of antiovarian autoantibodies (AOA) has been illustrated to be the convincing evidence for the diagnosis of autoimmune oophoritis^[8]^ as well as likely autoimmune POI.^[56]^ In patients with Addison’s disease, steroid cell antibodies (SCA) were found to be an autoantibody targeting ovary (theca interna and corpus luteum) and some other steroid-producing cells.^[8]^ According to reports, autoimmune oophoritis is related to various steroidogenic enzymes antigens, such as 17α-hydroxylase (17αOH), cytochrome P450 side chain cleavage enzyme (P450scc), 21-hydroxylase (21OH), and 3β-hydroxysteroid dehydrogenase (3β-HSD).^[50,57]^ Both 17αOH and P450scc, presenting in adrenal and ovaries, are major targets of SCA and are closely associated with POI.^[58]^ Over 90% of SCA-positive women are positive for the antibodies to 17αOH and/or P450scc.^[47]^ In contrast, 21OH is exclusively expressed in adrenal cortex, and its antibodies are associated with isolated Addison’s disease.^[58,59]^ Interestingly, with the absence of 21OH, less than 0.5% of women with POI are positive for these SCA. As a result, detection of 21OH autoantibodies in idiopathic POI patients has been recommended by European Society of Human Reproduction and Embryology guidelines.^[47,60]^

Nevertheless, the prevalence of 3β-HSD autoantibodies is controversial. Some researchers have found that autoantibodies against 3β-HSD could be detected in approximately 21% of women with isolated idiopathic POI, making it a major autoantigen of isolated POI.^[61]^ Controversially, other studies illustrated that although present in patients with Addison’s disease-related POI, 3β-HSD autoantibodies are sporadically positive in POI patients.^[57,62]^ The discrepancies in the prevalence of autoantibodies against 3β-HSD in different studies could be explained by different laboratory techniques. Besides, only 3% of patients with isolated POI are positive for at least one of three major immune markers of SCA (17αOH, P450scc and 3β-HSD).^[62]^ The clinical application of these markers is limited because of its low diagnostic sensitivity.

Zona pellucida,^[63]^ NACHT leucine-rich-repeat protein 5/maternal antigen that embryo requires,^[64]^ alpha-enolase,^[65]^ FSH^[66–68]^ and luteinizing hormone receptors^[69]^ are other candidate autoantigens, some of which could explain the clinical features of POI. Despite AOA are common in POI, their specificity and pathogenic effects are still questionable. More importantly, the presence of these antibodies in serum might not contribute to the occurrence and clinical activity of POI,^[70]^ which may be just the consequence of the disease rather than the cause. Further well-designed researches with larger samples are still needed.

### 4.2. Immunological disturbances in POI

Due to the increase of circulating autoantibodies and the presence of lymphocytic oophoritis, both cellular and humoral immunity play a critical role in the etiology of POI. More specifically, various literatures have described the immunological disturbances of POI patients, including immune cell, autoantibodies and cytokine profiles, which provide potent evidences for the association between immune disorder and POI.

#### 4.2..1. Immune cell profiles of POI patients.

T lymphocytes are the most important effectors in the immune pathogenesis of POI. It should be noted that due to the existence of different subsets, T lymphocytes could not only serve as inducers of autoimmune diseases, but also inhibit them.^[71]^ The proportion changes of T lymphocytes subsets have been widely reported and extremely suspected to be related with the occurrence and development of POI. Interestingly, although it has received countless attention, the conclusions on changes of the subsets are still inconsistent.

Multiple studies have revealed that the proportion of CD4^+^ T lymphocytes (CD4^+^ cells), a pro-inflammary T lymphocytes, were elevated in the peripheral blood of POI patients,^[72–75]^ implying that the enhanced inflammation status might be the risk factors POI. In theory, the potential autoreactive ability of CD4^+^ cells could be suppressed by regulatory T (Treg) cells, a subset of anti-inflammation T lymphocytes, to maintain the balance between pro-inflammation and anti-inflammation for a healthy immune system. However, decreased Treg cells and increased activated CD4^+^ cells in POI were reported,^[72]^ indicating a dysregulation of autoimmune reactions in POI. The underlying explanation might be the instability of Treg cells, which involves in the pathogenesis of this autoimmune disease.^[71]^ Besides, the mutation of forkhead box protein 3, also a significant regulator of Treg cells, could lead to the dysfunction of Treg cells, which would further result in multiple autoimmune disorders.^[76]^ Corresponding with the functional insufficiency of Treg cells on immune suppression, the mRNA levels of the forkhead box protein 3 transcriptional factor in the peripheral blood of POI patients were significantly decreased.^[77]^ These evidences indicated that when exploring the correlation between CD4^+^ cells and POI, both proportion and activation status of CD4^+^ cells (such as Treg cells counts) should be taken into account. For example, a prospective controlled trial showed that no change of CD4 + cells level was observed in the patients with idiopathic POI, although percentage of Treg cells significantly decreased, which was interpreted as the mechanism contributing to the autoimmune response in association with the presence of AOA.^[78]^ In this regard, although some other studies observed decreased^[79]^ or normal^[80–83]^ levels of CD4^+^ cells in POI, given that the detection of their activation status and Treg cells were lacking, the credibility of these studies might be limited.

Considering that the detection for at least two subsets of T lymphocytes might be required to truly reflect the immune status of POI patients, the ratio of CD4^+^ to CD8^+^ T lymphocytes (CD4^+^/CD8^+^) was tested by multiple researches. Interestingly, the percentage of CD8^+^ cells was found to be negatively related to the estradiol level of patients. Ho et al provided a negative correlation between plasma estradiol levels and CD8^+^ cells counts, and positive correlation between plasma estradiol levels and CD4^+^/CD8^+^ value.^[75]^ More importantly, lower CD4^+^/CD8^+^ value and higher CD8^+^ cells counts were also presented in patients with gonadal dysgenesis or hypothalamic-pituitary failure.^[84]^ These two studies implied that the changes in CD4^+^/CD8^+^ value and CD8^+^ cells counts might be attributed to estrogen deficiency. Furthermore, following two years of estrogen replacement, the percentage of CD8^+^ cells in idiopathic POI decreased significantly, resulting in an increase of CD4^+^/CD8^+^ value.^[85]^ This finding was consistent with another study in which lower CD4^+^/CD8^+^ value in POI patients could be partially reversed by hormone replacement therapy (HRT).^[79]^ The above results suggested that low CD4^+^/CD8^+^ value and high CD8^+^ cells counts seem to be a characteristic of POI, although it is still unknown whether they are causes or effects of POI. Nevertheless, these results remain controversial. The stabilization of CD8^+^ cells in POI was found in several studies,^[72,73,78–82]^ and a minority of studies showed declined^[83,86]^ percentage of CD8^+^ cells. Moreover, an elevated CD4^+^/CD8^+^ value in the peripheral blood of POI patients was reported by some studies.^[73,80,86]^ Although these findings are contradictory, the data support the important role of T lymphocyte-related injury in the pathogenesis of autoimmune POI.

Additionally, other immune cells also play critical roles in the pathology of POI. Various studies have found that peripheral blood CD19^+^ B cells, including CD5^+^ CD19^+^ B cells, the major source of natural antibodies and contributor to several autoimmune conditions,^[87]^ were markedly increased in POI patients.^[75,78,82,86,88]^ Nevertheless, there were a few exceptions. Mignot et al found that B lymphocyte counts, as well as immunoglobulins (IgG, IgA, IgE, and IgM) levels, in POI patients did not exceed those of healthy controls.^[73]^ Chernyshov et al reported unexpectedly that the numbers of CD19^+^ cells were even lower in POI patients, along with stable levels of CD5^+^ CD19^+^ cells.^[83]^

The polarization ability of monocytes in peripheral blood of POI patients was weakened under the influence of chemoattractant N-formyl-methionyl-leucyl-phenylalanine, and the capability of dendritic cells (DCs) prepared from peripheral blood monocytes to form clusters with allogeneic lymphocytes was also impaired in POI patients.^[89]^ Interestingly, similar to the reduction of Treg cells, considering that the changes of the gonadotropin and estrogen profile had no effect in this study, and postmenopausal women with the same hormone profile as POI patients did not show such defective functions, these defects of monocytes and DCs could not be ascribed to the hypergonadotropic hypoestrogenic hormone profile of POI patients. The possible reason of these abnormalities may be the redistribution of active monocytes and DCs from the periphery to the ovaries in POI patients.^[89]^

Taken together, it is suggested that the proportion and activity of immune cells should be comprehensively considered in the screen and diagnosis of autoimmune POI. Meanwhile, the combined detection of multiple immune cells could also be recommended. These efforts will contribute to more accurate strategies of early diagnosis and treatment of autoimmune POI in the future.

#### 4.2..2. Cytokine profiles of POI patients.

In addition to immune cells, some cytokines that contribute to the physiological functions of the ovary (as shown in Table 2) are also important candidates for the etiology of POI. Tumor necrosis factor-α (TNF-α), one of the most studied POI-related cytokines, is the major mediators of inflammatory responses.^[117]^ Moderate levels and proper functions of TNF-α are presumably necessary for normal ovarian function (Table 2). Previous studies based on rat models found that increased TNF-α could result in ovarian cell apoptosis and thereby accelerate follicular atresia,^[96,110,118]^ laying the pathological foundation for POI. However, whether high-level TNF-α could induce normal ovarian cell apoptosis in human remains unclear. Interestingly, studies have described lower levels of TNF-α in the serum of POI women, which might be attributed to diminished ovarian reserve, considering that TNF-α is also synthesized by oocyte and granulosa-luteal cells.^[119]^

**Table 2:** The involvement of various cytokines in physiological process in ovary.

Physiological process	Article	Species	Cytokines
Follicular/embryo development	Takehara et al (1994)^[90]^	Rabbit.	IL-1β
	Liu et al (2009)^[91]^	Mouse.	IL-6
	Goto et al (1997),^[92]^ Bornstein et al (2004),^[93]^ and Huang et al (2017)^[94]^	Human.	IL-8
	Gazvani et al (2000)^[95]^	Human.	IL-12
	Morrison et al (2002),^[96]^ Ma et al (2010)^[97]^ Mendoza (1999) et al,^[98]^ Lee et al,^[99]^ and Spaczynski et al (1999)^[100]^	Mouse, human, pig, Human, and rat.	TNF-α
Ovulation	Gérard (2004) et al^[101]^ and Vinatier et al (1995)^[102]^	Human.	IL-1
	Gallinelli et al (2003)^[103]^	Human.	IL-2
	Chatterjee (2020) et al,^[104]^ Geva (1997) et al,^[105]^ and Gonzalez et al (2017)^[106]^	Anabas testudineus, human, and human.	IL-6
	Bornstein (2004) et al^[93]^ and Runesson et al (1996)^[107]^	Human.	IL-8
	Gonzalez et al (2018)^[106]^	Human.	IL-10
	Tsuji et al (2001)^[108]^	Mouse.	IL-18
	Carlock et al (2014)^[109]^	Mouse.	IL-33
	Yamamoto et al (2015),^[110]^ Vinatier et al (1995),^[102]^ Gonzalez et al (2018),^[106]^ Brännström et al (1995),^[111]^ and Williams et al (2008)^[112]^	Mouse, human, human, Rat, and bovine.	TNF-α
Corpus luteum formation and regression	Galvão et al (2013)^[113]^	Rat.	IL-1
	Sakumoto et al (2006)^[114]^	Pig.	IL-4
	Bornstein et al (2004),^[93]^ Gonzalez et al (2018),^[106]^ and Sakumoto et al (2006)^[114]^	Human, human, and pig.	IL-6
	Bornstein et al (2004)^[93]^ and Galvão et al (2013)^[113]^	Human and rat.	IL-8
	Gonzalez et al (2018)^[106]^ and Yang et al (2019)^[115]^	Human.	IL-10
	Gonzalez et al (2018)^[106]^ and Galvão et al (2013)^[113]^	Human and rat.	TNF-α
	Yang et al (2019)^[115]^	Human.	IFN-γ
Steroidogenesis	Gérard et al (2004)^[101]^ and Popovic et al (2019)^[116]^ Gallinelli et al (2003)^[103]^	Human and hamster. Human.	IL-1 IL-2
	Sakumoto et al (2006)^[114]^	Pig.	IL-4
	Gonzalez et al (2018)^[106]^ and Sakumoto et al (2006)^[114]^	Human and pig.	IL-6
	Bornstein et al (2004)^[93]^ and Gallinelli et al (2003)^[103]^	Human.	IL-8
	Gonzalez et al (2018)^[106]^	Human.	IL-10
	Gallinelli et al (2003)^[103]^	Human.	IL-11
	Morrison et al (2002),^[96]^ Gonzalez et al (2018),^[106]^ and Williams et al (2008)^[112]^	Mouse, human, and bovine.	TNF-α

IFN-γ = interferon-γ, IL = interleukin-29, TNF-α = tumor necrosis factor-α.

It has been reported that TNF-α promoter polymorphisms −1031C, −308A, and −238A could increase the risks of POI in Korean women.^[120]^ TNF-α works through two types of receptors: TNF type I receptor and TNFR2, which contribute to cell apoptosis and proliferation, respectively.^[120]^ It was observed that the concentration of soluble TNFR2 is closely related to TNF-α, and exert regulatory properties on the activity of TNF-α by binding to TNF-α and thus preventing the ligand from interacting with cell surface TNFR2. In a prospective study, a non-linear association of soluble TNFR2 with early menopause was observed. More specifically, compared to women with lower or higher circulating levels of soluble TNFR2, the risks of early menopause were decreased in women with moderate levels.^[121]^

Recently, Xiong et al revealed that the serum level of pro-inflammatory interferon-*γ* (IFN-*γ*) was elevated, whilst the level of anti-inflammatory Transforming growth factor *β* (TGF-*β*) was decreased in POI patients.^[77]^ TGF-*β* inhibits the differentiation of cytotoxic T cells, T help 1 (Th1) and Th2 cells, and promotes the generation of peripheral Treg and Th17 cells. Meanwhile, TGF-*β* regulates the proliferation, survival, activation and differentiation of B cells, as well as the development and function of NK cells, macrophages, DCs and granulocytes. Studies based on rodent models have found that TGF-*β* injection could inhibit the development of several autoimmune diseases, such as T1DM, multiple sclerosis and SLE.^[122]^ Moreover, the levels of TGF-β were found to inversely associated with disease severity of the patients with multiple sclerosis or SLE.^[122]^ Therefore, it is reasonable that the decreased levels of TGF-β could be observed in POI patients. Preclinical and clinical trials for POI therapy using TGF-β-related treatments are expected in the future.

IFN-γ not only has antiviral capacity, but also presents at abnormally high levels in lymphocytes from patients with multiple sclerosis.^[123]^ In a mouse model, T cells expressing IFN-γ were found to infiltrate into islets, leading to destruction of beta cells and T1DM.^[123]^ In abnormal immune circumstances, IFN-γ is secreted by Th1 cells whose proliferation is inhibited by Treg cells.^[77]^ Therefore, combined with aforementioned Treg cell profile in POI patients, the reduction of Treg cells may alleviate the inhibition of IFN-γ production and thereby becomes insufficient to maintain immune tolerance, which might be ascribed to the lower levels of TGF-β. In addition, interleukin (IL)-29, also known as IFN-λ1 belonging to the IFN family, possesses antiviral activity and modulatory functions in immune responses.^[124]^ It was found that IL-29 was downregulated in POI, suggesting that the reduction of IL-29 promotes the autoimmune response and contributes to the progress of POI.^[125]^ Given that the regulatory network of immune balance is bidirectional and intricate, the exact mechanisms should be explored by more basic researches.

To date, several ILs have been reported to be changed in POI. Sun et al found that the serum concentration of IL-6 and IL-21 significantly increased in POI patients with immune abnormalities, and were negatively related to ovarian reserve.^[126]^ The IL-6/IL-6R complex plays an immunomodulatory role in multiple processes of immune responses by regulating lymphocyte differentiation (such as disrupting Th17/Treg balance) and promoting antibody secretion.^[127]^ In addition, IL-21 has multifarious effects on both humoral and cellular immune responses, such as enhancing lymphocyte proliferation, NK cell function and the differentiation of B cells into plasma cells.^[128]^ These results implied the potential effects of IL-6 and IL-21 on autoimmune POI. However, the mechanisms of these two cytokines influencing patients with autoimmune POI still need more basic experiments for illustration.

IL-17F and IL-17C interacting with IL-17R constitute an important part of immune system, and contribute to the pathology of various inflammatory disorders.^[129,130]^ Liu et al found that IL-17 members, including IL-17F, IL-17C and IL-17R, were decreased in POI patients, suggesting the complex roles of IL-17 members in POI.^[125]^ Although lack of studies discovering the involvement of IL-2 in POI, the increased expression of IL-2 receptors on peripheral T lymphocytes in some patients with spontaneous POI may reflect the activation of peripheral T lymphocytes. Additionally, elevated soluble IL-2 receptor levels in body fluids are associated with increased T and B cell activation and immune system activation. However, no significant difference of serum soluble IL-2 receptor was detected between POI and normal subjects.^[131]^

Furthermore, the roles of pro-inflammatory IL-1α and IL-1β in ovarian reserve were illustrated by Uri-Belapolsky et al^[132]^ using transgenic mice models. Although statistical significance of increased IL-1β level was absent, the level of IL-1α was remarkably higher in both serum and follicular fluid (FF) of POI patients,^[133]^ indicating the close association between IL-1α and immune mechanisms of POI. Interestingly, the levels of both IL-1α and IL-1β in FF were significantly elevated than those in serum,^[133]^ highlighting the underlying importance of immune imbalance in follicular microenvironment in the pathogenesis of POI.

However, another recent study evaluated the cytokine profile in biochemical POI (bPOI) patients, and drew inconsistent conclusions.^[134]^ In this study, a total of 45 cytokines were detected. Except for higher levels of IL-27, IL-7, and IL-17A in FF and a lower level of IL-1α and IL-21 in serum of bPOI women, nearly all ILs mentioned above, in addition to TNF-α and IFN-γ, were extremely low and showed no difference between bPOI and controls. And the cytokine characteristics in the paired serum were not accordant with those in FF between the two groups. It is widely accepted that follicular microenvironment is important for folliculogenesis and acquisition of oocyte competence. The discrepancy could be partially explained by the novel testing method, magnetic beads multiplex immunoassay, used in this study, whose sensitivity might be low.

In consequence, a series of cytokines in FF have been demonstrated to be involved in various physiological functions of ovary, and immune disturbances in follicular microenvironment may have serious adverse effects on ovaries. The cytokine profile in FF might be more meaningful than those in circulation of POI patients, which requires larger samples and multicenter studies.

## 5. The management of autoimmune POI

The development of POI can be divided into four stages: normal, latent, biochemical and clinical abnormality.^[126]^ Due to the latency of POI in early stages, when symptoms are obvious, patients tend to have been in advanced stages, making treatment less efficient. Therefore, early diagnosis is the key for timely treatment and good prognosis of POI. It is assumed that autoimmune POI should be identified by the coexistence of ovarian histological evidences and serum autoantibodies against ovary, adrenal cortex and/or steroid-producing cells, as well as the concurrence of autoimmune diseases, such as autoimmune Addison’s disease and thyroid disorders. Ovarian biopsy is the gold standard for detecting autoimmune involvement of ovarian tissue and the presence of developing follicles. Considering the invasive and expensive characteristics of ovarian biopsy, a noninvasive screening approach is required to bypass unnecessary ovarian biopsy and subsequent ovarian damage.^[3]^ Recently, positivity of 21 OH, side-chain cleavage enzyme (SCC), 17 OH and NALP-5 has been perceived as diagnostic evidence for autoimmune POI in two international multi-center cohorts.^[56]^

According to the European Society of Human Reproduction and Embryology guideline, the following investigations should be recommended to patients with suspected POI^[60]^:

- Chromosomal analysis in women with non-iatrogenic POI.
- Fragile-X premutation testing in POI women. Autosomal genetic testing is not indicated, unless a specific mutation (e.g., blepharophimosis–ptosis–epicanthus inversus syndrome) is suggested.
- Screening for 21OH antibodies (or alternatively adrenocortical antibodies) in POI women with unknown cause or if an immune disorder is suspected; If positive, adrenal function testing is referred to rule out Addison’s disease.
- Screening for thyroid antibodies in POI women with unknown cause or if an immune disorder is suspected; If positive, thyroid stimulating hormone should be measured annually.
- Routine screening POI women for diabetes or infection is not recommended.
- The possibility of POI as a consequence of a medical or surgical intervention should be discussed.

The treatment of autoimmune POI is sophisticated and requires multidisciplinary management, mainly including two main aspects: HRT and infertility therapy. Considering menopausal symptoms and long-term complications related to estrogen deprivation, HRT is recommended for the treatment of POI, which may be important for primary prevention of cardiovascular diseases and osteoporosis.^[2,135,136]^ Although there are still chances of spontaneous pregnancy for POI women, the odds is extremely low with 5%.^[4]^ Recently, in vitro activation of dormant follicles and in vitro maturation of immature oocytes have been emerging.^[4,137,138]^ Cryopreservation of oocytes, embryos, and ovarian tissue seems to be a feasible method of fertility preservation, but the technical problems impede the development of the oocyte cryopreservation because of its susceptibility to cryoinjury. To date, ovarian tissue cryopreservation and transplantation is still at the experimental stage.^[139]^ Oocyte donation is another choice for POI patients to conceive.^[3,50]^ Emotional support is also of importance for these patients to deal with infertility.

Moreover, the lymphocytic infiltration of developing follicles mainly exists in primary and primordial follicles, providing the possibility of immunosuppressive treatments for ovarian function resumption. Corticosteroid administration may restore ovarian function, whereas the use of immunosuppressive agent is controversial,^[140,141]^ and the potential serious complications caused by these drugs should also be considered. Prospective, randomized and controlled studies are required to draw more precise conclusions. Dehydroepiandrosterone (DHEA) supplementation may increase AFC and ovarian volume of POI women,^[142]^ and increase the pregnancy rates in patients with diminished ovarian reserve.^[143]^ In addition, a proportion of POI cases with Hashimoto’s thyroiditis benefit from DHEA sulfate (DHEAS) supplementation.^[144]^

These data suggested that DHEA and/or DHEAS supplementation may serve as a potentially useful therapeutic strategy for POI. With the potential of self-renewal and regeneration, stem cells could also be utilized as a possibly effective treatment for ovarian failure and consequent infertility.^[145]^ Among numerous stem cells, the effects of human placenta mesenchymal stem cells, human umbilical cord-derived mesenchymal stem cells (hUCMSCs) and human amniotic epithelial cells transplantation have been confirmed to attenuate autoimmune POI in mice.^[146–148]^ Encouragingly, two clinical trials from China suggested that the transplantation of hUCMSCs could significantly improve follicular growth and rescue ovarian function in women with POI.^[149,150]^ Although only a few clinical deliveries were obtained from POI patients after hUCMSC transplantation, all of these babies are developed normally.^[149]^ Consequently, stem-cell techniques might open a new field toward the treatment of POI, and more clinical trials with large samples from different countries or regions are expected.

## 6. Conclusion

In conclusion, this review summarized the immune profiles of patients with autoimmune POI, and described the relationship of autoimmunity with the diminished ovarian reserve and POI. Furthermore, the correlation of ovarian reserve with various autoimmune disorders was elucidated, and the evidences for the possibility of POI etiological were provided. Immune system, principally including inflammatory cells (lymphocytes, macrophages, and DCs) and cytokines (such as IL-1, IL-2, IL-6, IL-8, and TNF-α), participates in several aspects of ovarian physiological function, and plays a crucial role in the development of autoimmune POI. In addition, possibly feasible treatment of autoimmune POI was discussed, whilst further inspiring novel therapies for autoimmune POI are expected.

## Author contributions

**Project administration:** Manhua Cui.

**Writing – original draft:** Junyu Chen, Shan Wu.

**Writing – review & editing:** Mengqi Wang, Haoxian Zhang.
